# Defining the optimal treatment strategy for localized prostate cancer patients: a survey of ongoing studies at the National Cancer Institute of Canada Clinical Trials Group

**DOI:** 10.3747/co.v15i4.257

**Published:** 2008-08

**Authors:** W.R. Parulekar, M. McKenzie, K.N. Chi, L. Klotz, C. Catton, M. Brundage, K. Ding, A. Hiltz, R. Meyer, F. Saad

**Affiliations:** * National Cancer Institute of Canada Clinical Trials Group, Kingston, ON.; † BC Cancer Agency, Vancouver, BC.; ‡ Sunnybrook Health Sciences Centre, Toronto, ON.; § Princess Margaret Hospital, Toronto, ON.; || Cancer Centre of Southeastern Ontario at Kingston General Hospital, Kingston, ON.; # CHUM–Hôpital Notre Dame, Montreal, QC.

**Keywords:** Localized prostate cancer, risk estimation, clinical trials

## Abstract

The designation “clinically localized prostate cancer” comprises a group of biologically heterogeneous tumours with different growth rates and risks of relapse. Because prostate cancer is primarily a disease of older men, treatment selection must take into account the prognosis of the tumour, patient age, comorbidities, side effects of treatment, and patient preferences. Clinical trials must identify the various prognostic groups and test the appropriate treatment strategies within these subgroups.

## 1. INTRODUCTION

Prostate cancer is the most common malignancy in Canadian men and ranks third behind lung and colon cancer in terms of cancer-related mortality. However, from 1994 to 2003, mortality from prostate cancer declined at a rate of 2.7% annually. That decline is attributed both to the widespread use of testing for prostate-specific antigen (psa), which has led to a shift in stage and grade at diagnosis, and to the existence of effective therapies for clinically localized disease.

In 2007, estimates placed new cases of prostate cancer at 22,300 and deaths from the disease at 4300 1. Those statistics highlight some important facts about prostate cancer:

- In most cases, prostate cancer is not a fatal condition.
- Current treatment options still fail to cure or control disease in a significant proportion of cases, and approximately 20% of patients die from their prostate cancer.

Not surprisingly, the treatment strategies under evaluation in ongoing clinical trials in early prostate cancer reflect the biologic heterogeneity of the disease. They include such diverse therapies as active surveillance for good-prognosis disease and the addition of cytotoxic chemotherapy to radical radiation or prostatectomy for disease with high risk of relapse. The present article reviews ongoing studies in localized prostate cancer conducted by the National Cancer Institute of Canada (ncic) Clinical Trials Group (ctg).

## 2. NCIC CTG PR.11: A PHASE III STUDY OF ACTIVE SURVEILLANCE THERAPY AGAINST RADICAL TREATMENT IN PATIENTS DIAGNOSED WITH FAVOURABLE-RISK PROSTATE CANCER (START)

### 2.1 Background

Tumours detected because of psa testing comprise most of the localized prostate cancer cases diagnosed today. Although testing may allow for diagnosis and the use of curative therapy at an earlier stage in a potentially life-threatening disease, it also clearly identifies a group of patients with biologically indolent tumours in whom radical therapy may be unnecessary and detrimental because of its associated morbidity and costs 2,3. Previous nonrandomized studies have identified the prognostic significance of stage and grade in patients treated with conservative therapy or observation, thus identifying a patient population for whom curative therapy can potentially be withheld without compromise to long-term outcome 4–6. An extension of the concept of observation is that of “active surveillance,” which entails close follow-up of disease and intervention with curative intent triggered by early signs of disease progression.

The strategy of active surveillance for patients with favourable-risk prostate cancer was evaluated in a large phase ii study by Klotz. Active surveillance was applied in 331 patients with favourable-risk disease (defined as psa below 15 ng/mL, Gleason score of 7 or less, and tumour stage less than T2B), following them until criteria of early disease progression [defined by biochemical, histologic (grade), or clinical progression] were met. Of those patients, 80% had a Gleason score of 6 or less, and the same proportion had a psa below 10 ng/mL. With a median follow-up of 72 months, 34% of patients discontinued active surveillance. Biochemical progression led to discontinuation in 15%; clinical progression, in 3%; histologic progression, in 4%; and patient preference, in 12%. With a median follow-up of 7 years, overall survival was 85%, and disease-specific survival was 99%. The median psa doubling time for the entire cohort was 7.0 years; a psa doubling time of less than 2 years was associated with a high risk of local progression for patients who underwent radical prostatectomy. At January 2007, 134 patients remained on active surveillance 7.

### 2.2 Study Design

The PR.11 trial is an Intergroup study led by the ncic ctg (study chair, Dr. Laurence Klotz) that compares active surveillance with radical therapy (prostatectomy or radiotherapy depending on physician and patient choice) at the time of diagnosis in a randomized phase iii setting. Eligible patients are those with a life expectancy of more than 10 years and favourable-risk prostate cancer [defined as clinical stage T1B, T1C, T2A, or T2B at the time of diagnosis; clinical (diagnostic biopsy) Gleason score of 6 or less; psa 10.0 ng/mL or lower]. Patients randomized to the active surveillance arm will undergo radical intervention (again, prostatectomy or radiotherapy depending on physician and patient choice) at the time one or more of the following pre-specified criteria are met:

- Biochemical progression—psa doubling time less than 3 years, based on at least 5 separate consecutive measurements over a minimum of 12 months from the date of the baseline measurement or from the date that the psa reached a value greater than or equal to the psa before initiation of androgen deprivation therapy (if applicable), as assessed by the local investigator.
- Histologic or grade progression—Gleason pattern predominant 4 or greater (that is, a Gleason score of 7 (4+3) or higher) in re-biopsy of the prostate.
- Clinical progression—”local progression” defined as local progression of prostate cancer resulting in urinary retention, gross hematuria, or hydronephrosis; or “distant metastasis” defined by radiology, cytology, or histology (or a combination) at sites remote from the prostate and regional lymph nodes.

Using a non-inferiority design to rule out a greater than 5% difference in 15-year survival between the radical treatment and active surveillance groups, 2130 patients will be accrued over a 5-year period. The primary endpoint is disease-specific survival. That endpoint, rather than overall survival, was selected because of the need to determine the effect of the active surveillance strategy specifically on prostate cancer mortality. Secondary endpoints include overall survival, quality of life, distant disease-free survival, psa relapse or progression after radical intervention, initiation of androgen deprivation therapy, proportion of patients on the active surveillance arm receiving radical intervention, prognostic significance of psa doubling time before diagnosis, and prognostic significance of molecular biomarkers. Quality of life is an important part of the study, and the Expanded Prostate Cancer Index, rand SF-12, and State–Trait Anxiety Inventory will be used to provide a comprehensive examination of the various components of patient-reported outcomes on study. The feasibility phase has commenced in designated centres of participating cooperative groups. If the results of the feasibility phase indicate sufficient patient and physician willingness to participate in the randomization process, then accrual will be opened widely.

## 3. NCIC CTG PR.12: A PHASE III STUDY OF NEOADJUVANT DOCETAXEL AND ANDROGEN SUPPRESSION PLUS RADIATION THERAPY VERSUS ANDROGEN SUPPRESSION ALONE PLUS RADIATION THERAPY FOR HIGH-RISK LOCALIZED ADENOCARCINOMA OF THE PROSTATE (DART)

### 3.1 Background

Radical radiotherapy and long-term androgen suppression constitute an accepted treatment option for localized but high-risk disease as defined by clinical stage (T3) and high Gleason score (8 or higher) or high psa (20 ng/mL or more), or both. Results from previously conducted randomized studies are consistent with 5-year disease-free survival rates of 46%–74% with combined therapy 8–10, thus providing the rationale for continued evaluation of therapies to improve outcome by control of micrometastatic disease.

Docetaxel is a good candidate drug. The mechanism of action of this agent involves disruption of the microtubular network critical for mitotic and inter-phase cellular functions. Doses of 75–100 mg/m^2^ intravenously (IV) administered are well tolerated, with neutropenia, alopecia, cutaneous reactions, gastro-intestinal effects (nausea, diarrhea), neurotoxicity, and edema being among the most frequently reported adverse events. Severe hypersensitivity reactions characterized by respiratory or circulatory instability or generalized rash or erythema occur in fewer that 5% of patients, although lesser grades are more common 11. Using overall survival as the primary endpoint, two pivotal studies have demonstrated the efficacy of docetaxel in advanced hormone-refractory prostate cancer 12,13. Efficacy and adverse event data support the use of the every-three-weeks docetaxel schedule, and that schedule has been widely adopted for use in this patient population.

Using changes in psa as a marker of antitumour effect, studies have shown that docetaxel is also active against hormone-sensitive prostate cancer 14–17. Furthermore, based on preclinical data that suggest that docetaxel may result in phosphorylation and inactivation of the anti-apoptotic protein Bcl-2 (which is upregulated with androgen suppression), combination therapy with docetaxel and androgen suppression may lead to greater antitumour effect 18–21.

Timing of therapy appears to be important. Eigl *et al.* implanted LNCaP human prostate cancer and Shionogi mouse mammary carcinoma cell lines into mice, and followed up with treatment using one of these three regimens: castration with paclitaxel on progression, paclitaxel with castration on progression, or concurrent castration and paclitaxel 22. As compared with sequential castration followed by paclitaxel, concurrent therapy resulted in significantly longer time-to-progression and time-to-sacrifice in the mice. Notably, a marked lack of response to castration in the mice treated initially with paclitaxel was seen.

Clinical studies in patients with locally advanced prostate cancer have demonstrated the feasibility and tolerability of combined therapy with docetaxel and androgen suppression in the neoadjuvant setting before prostatectomy 23 or radiotherapy 24. In 54 men with high-risk prostate cancer, McKenzie *et al.* used one of two neoadjuvant treatment schedules before radical radiotherapy: 6 months of androgen suppression, plus 2 cycles of docetaxel 35 mg/m^2^ IV weekly for 6 weeks out of 8; or 5 months of androgen suppression, plus 4 cycles of docetaxel 75mg/m^2^ IV every 3 weeks. Androgen suppression was continued after completion of radiotherapy for a total duration of 3 years. The primary endpoint was unacceptable toxicity. Eight patients (14.8%) developed unacceptable toxicity: 5 in the weekly docetaxel regimen [grade 3 acute genitourinary radiotherapy-related adverse events (*n* = 3), grade 3 docetaxel hypersensitivity (*n* = 1), grade 3 fatigue lasting more than 2 weeks (*n* = 1)] and 3 in the every-three-weeks arm [febrile neutropenia (*n* = 1), grade 4 neutropenia lasting more than 7 days, grade 3 acute genitourinary radiotherapy-related adverse event (*n* = 1)]. Compliance with the radiotherapy was excellent, and all patients completed planned treatment. Long-term follow-up continues. The neoadjuvant regimen containing androgen suppression and every-three-weeks docetaxel was chosen for further study based on the promising results of this pilot and the proven efficacy and tolerability of every-three-weeks docetaxel dosing in the advanced-disease setting.

### 3.2 Study Design

The ncic ctg PR.12 trial is a phase iii study comparing the every-three-weeks docetaxel and neoadjuvant androgen suppression regimen piloted by McKenzie *et al.* to androgen suppression alone in addition to radical radiotherapy (three-dimensional conformal radiotherapy, 46 Gy in 23 fractions, with 24–28 Gy in 12–14 fractions). Study chairs are Drs. Michael McKenzie and Kim Chi. In both treatment arms, androgen suppression will be given for a total duration of 3 years. Patients with high-risk disease (defined as at least clinical stage T3 or T4, Gleason score of 8 or higher, or psa above 20 ng/mL) are eligible for the study. The primary endpoint is disease-free survival. The sample size for this study is estimated based on detecting an estimated 33.3% risk reduction in disease progression favouring the experimental arm [hazard ratio (hr): 0.667], using a 1-sided log-rank test at the 2.5% significance level and 90% power. An estimated 530 patients (assuming a 14.8% loss to follow-up) will be accrued over 4.5 years, with an additional 5 years of follow-up. Secondary endpoints include overall survival, time to biochemical disease progression, time to local disease progression, time to distant disease progression, time to next anticancer therapy, progression-free survival, degree of psa suppression before radiotherapy, quality of life, and adverse events. Centres will be credentialed by ncic ctg for delivery of radiotherapy before randomization of the first patient. Tumour and biologic specimens will be collected during the study to determine the prognostic role of cytokines and insulin-like growth factor axis markers. In addition, cytokine levels and changes in levels over time will be correlated with fatigue (as measured by the Common Terminology Criteria, version 3.0) and quality of life.

## 4. NCIC CTG PR.13: RADIOTHERAPY AND ANDROGEN DEPRIVATION IN COMBINATION AFTER LOCAL SURGERY (RADICALS)

### 4.1 Background

The PR.13 study represents a collaborative effort between the Medical Research Council Clinical Trials Unit (United Kingdom) and the ncic ctg (Canadian study chairs: Drs. Charles Catton and Fred Saad). This large pragmatic study is addressing two fundamental issues in the postoperative management of patients with resectable prostate cancer: What is the optimal timing of radiotherapy in these patients? And what role, if any, does androgen suppression play in determining outcome? The relevance of the study to current practice is underscored by the fact that prostatectomy is a standard of care in men presenting with operable prostate cancer. In Ontario alone, the number of radical prostatectomies between 1993–1994 and 2003–2004 rose by 171% 25.

The role of postoperative radiotherapy has been addressed in three randomized studies:

- In eortc 22911, 1005 patients with pT3 disease post radical prostatectomy were randomized to either observation or adjuvant radiotherapy 26. The primary endpoint, local control, was modified to clinical progression-free survival and later to biochemical progression-free survival. After a median follow-up of 5 years, biochemical progression-free survival was significantly improved in the irradiated group [74.0%; 98% confidence interval (ci): 68.7 to 79.3] as compared with the observation group (52.6%; 98% ci: 46.6 to 58.5; *p* < 0.0001). Clinical progression-free survival was significantly better with adjuvant radiation (hr: 0.61; 98% ci: 0.43 to 0.87; *p* = 0.0009). No difference in overall survival was detected. The rate of 5-year grade 3 or higher toxic effects was 2.6% in the no-further-treatment group and 4.2% in the postoperative irradiation group (*p* = 0.0726). The incidence of grade 3 urethral stricture and incontinence was 1.4% (6 patients) in each group.
- A similar design was used in swog 8794 (ncic ctg PR.2), which randomized 425 men with pT3 disease to observation or to radiotherapy to the prostate bed 27. The primary endpoint was metastasis-free survival, defined as the time from randomization to first evidence of metastatic disease or death from any cause. With a median follow-up of 10.6 years, the metastases-free survival was not significantly different between the two arms (hr: 0.75; 95% ci: 0.55 to 1.02; *p* = 0.06). Overall survival favoured the adjuvant radiotherapy arm, but did not reach statistical significance (hr: 0.80; 95% ci: 0.58 to 1.09; *p* = 0.16). The rate of biochemical relapse was significantly lower in men with an undetectable psa level post prostatectomy (*n* = 249) treated with adjuvant radiotherapy (hr: 0.43; 95% ci: 0.31 to 0.58; *p* < 0.001), as was recurrence-free survival [defined as survival without evidence of measurable or evaluable disease, excluding psa relapse (hr: 0.62; 95% ci: 0.46 to 0.82; *p* = 0.001)]. Approximately one third of patients randomized to the observation arm ultimately received pelvic radiotherapy. Rectal complications (3.3% vs. 0%, *p* = 0.02), urethral stricture [17.8% VS. 9.5%; risk ratio (rr): 1.9; 95% ci: 1.1 to 3.1; *p* = 0.02), and urinary incontinence (6.5% vs. 2.8%; rr: 2.3; 95% ci: 0.9 to 5.9; *p* = 0.11) were more frequent in the adjuvant radiotherapy arm.
- Results from the aro 96–02 study were reported at the 2007 meeting of the American Society of Clinical Oncology 28. That study randomized patients with pT3 disease to adjuvant radiotherapy or a “wait-and-see policy.” Those who failed to achieve an undetectable psa level postoperatively on either arm were given a designation of progressive disease and offered radiotherapy. The primary endpoint, biochemical control, was significantly improved in the adjuvant radiotherapy arm (hr: 0.53, *p* = 0.0015).

Taken together, the results of the foregoing trials fail to fully inform physicians and patients about the role of post-prostatectomy radiotherapy in current practice because of differences in outcome definitions used in the trials, lack of consistent effect of adjuvant radiotherapy on clinical (non-psa) endpoints, variable use of late radiotherapy in patients randomized to the observation arm, and current use of assays for psa testing that are more sensitive than those used during the studies.

The situation regarding the use of hormone therapy in this group of patients is even less clear. No randomized controlled trials have reported addressing the role and optimal duration of hormone therapy in men receiving post-prostatectomy radiotherapy. The uncertainty among clinicians regarding the role of adjuvant radiotherapy and hormone therapy is reflected in recent surveys of urologists and oncologists, indicating a wide variation in use of these therapies in the post-prostatectomy patient population 29,30.

### 4.1 Study Design

The radicals trial is designed to address the issues of radiotherapy timing (immediate vs. early salvage) and of hormone therapy duration (none vs. short-term vs. long-term). The primary endpoint is disease-specific survival. It is estimated that the radiotherapy timing randomization will have to recruit 2600 patients and the hormone-duration randomization, 3500 patients. Many patients will be in both randomizations. The trial is planned to address these questions over 12–13 years with 5.5 years of accrual and 7 years of further follow-up. Secondary endpoints include freedom from treatment failure, clinical progression-free survival, overall survival, non-protocol hormone therapy, treatment toxicity, and patient-reported outcomes.

The radiotherapy timing randomization involves immediate radiotherapy to the prostate bed versus a salvage radiotherapy policy at the time of psa failure. The radiotherapy will use standard techniques and dose fractionation schedules: 66 Gy in 33 fractions over 6.5 weeks or 52.5 Gy in 20 fractions over 4 weeks. The hormone duration randomization involves no hormone therapy with radiotherapy, compared with short-term (6 months) hormone therapy beginning shortly before radiotherapy, compared with long-term (24 months) hormone therapy beginning shortly before radiotherapy. Patients who decide not to enter the three-way randomization will be able to choose randomization between two of the three arms: 0 as compared with 6 months of hormone therapy if they do not want to be randomized to a long duration of treatment, or 6 as compared with 24 months of hormone therapy if they do not want to be randomized to the no-hormonetherapy treatment arm.

Key eligibility criteria for the radiotherapy timing randomization include a postoperative serum psa below 0.4 ng/mL within 3 months after radical prostatectomy, and uncertainty in the opinion of the clinician and patient regarding the need for immediate postoperative radiotherapy. For the hormone duration randomization, patients must be expected to receive radiotherapy (adjuvant or salvage) and must have a psa of 10 ng/mL or more at the time of randomization. In an 18-month feasibility stage, radicals will carefully assess randomization rates and the trial as a whole. Continuation of the trial beyond the feasibility stage will be conditional on satisfactory patient accrual.

## 5. SUMMARY

Ongoing studies at the ncic ctg are addressing fundamental questions regarding the management of localized prostate cancer.

The randomized phase iii Intergroup study PR.11 led by ncic ctg is asking the single most important question regarding the management of favourable-risk prostate cancer: Is active surveillance with a radical intervention based on signs of disease progression as good as radical intervention at diagnosis? The results of this study, whether positive or negative, have the potential to define the management of low-risk prostate cancer globally and to clarify the role of psa doubling time in decision-making.

The hypothesis being tested in PR.12 is whether the addition of docetaxel to standard treatment with androgen suppression combined with radiotherapy improves outcome in a high-risk prostate cancer population. This study builds on preclinical data demonstrating the interaction between taxanes, androgen suppression, and development of androgen resistance, and also the extensive literature demonstrating activity of docetaxel in prostate cancer.

Finally, PR.13 is a large study that seeks to clarify the roles of post-prostatectomy radiotherapy timing (adjuvant vs. relapse) and the optimal duration of hormone therapy (0 months vs. 6 months vs. 24 months) in patients already treated with prostatectomy.
